# Supplementary material for: Non-trivial relationship between scaling behavior and the spatial organization of GDP in Indonesian cities
Source: PLoS One. 2022 Nov 10;17(11):e0277433. doi: 10.1371/journal.pone.0277433 (PMC9648789; doi:10.1371/journal.pone.0277433)
Supplement: S1 Appendix — Contains all the supporting figures. (PDF) [file pone.0277433.s001.pdf]

S1 Appendix: Spatial organization of the population and the scaling exponents β

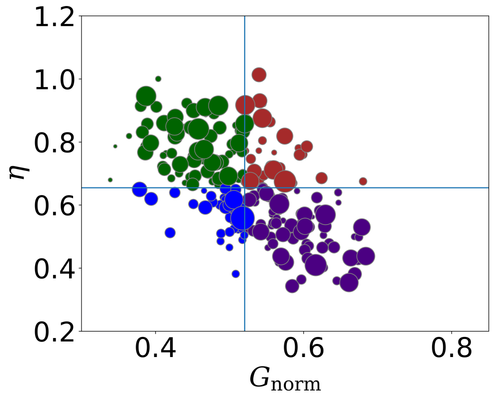

FIG. S1. **Classification of Indonesian cities based on the spatial organization of the population.** Spreading index of hotspots η versus normalized Gini coefficient G_{norm}, circle size represents population size, color represents the classifications. Green, brown, purple, and blue for Homo-Poly, Hete-Poly, Hete-Mono, and Homo-Mono classes, respectively.

The gridded GDP dataset [1] used in the main text is constructed from the gridded population dataset [2] and the sub-national GDP per capita data. To clarify how sub-national GDP per capita data plays a role in the spatial organization of the GDP, we repeat our analysis using the gridded population density and check the differences. We plot Indonesian cities in the plane of spreading index of hotspots η and normalized Gini coefficient G_{norm} in Fig. S2, which corresponds to Fig. 4A in the main manuscript. We observe that the negative correlation between η and G_{norm} still exists. However, one can see that, in general, lower G_{norm} is observed when we use population density data. This demonstrates the role of the GDP percapita prefactor in the gridded GDP dataset, where stronger inequalities takes place due to less populated grid has lower GDP per capita and vice versa. We then quantify the differences by calculating the Euclidean distance $d(\mathrm{GDP}, \mathrm{Pop})^2$ in the plane (G_{norm}, η) of each cities between the spatial organizations of the population (Fig. S2) and GDP (Fig. 4A) in Fig. S3. Here, one can see that small cities tend to differ more than the large counterpart.

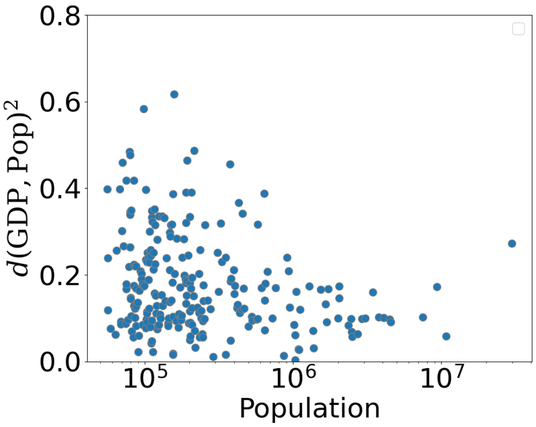

FIG. S2. **Differences between spatial organization of GDP and population of Indonesian ciites.** Plot of Euclidean distance $d(\mathrm{GDP}, \mathrm{Pop})^2$ in the plane (G_{norm}, η) against population.

The above differences yield different classifications. Thus, a different scaling behavior of each class is expected. We plot the scaling exponent of each class obtained from spatial organization of the population in Fig. S4, which corresponds to the lower half of Fig. 5 in the main text. Surprisingly, we find that between the data source, the scaling exponents are almost similar (always in the same scaling regime). It has one exception for the Hete-Poly class, where the commuting area scales superlinearly with the population, in contrast to the sublinear scaling in

the classification using spatial organization of the GDP. Note that the Hete-Poly class represents discontinuous or "leapfrog" development. Such differences might take place due to smaller cities with discontinuous development have a lot of grid with high population but low GDP.

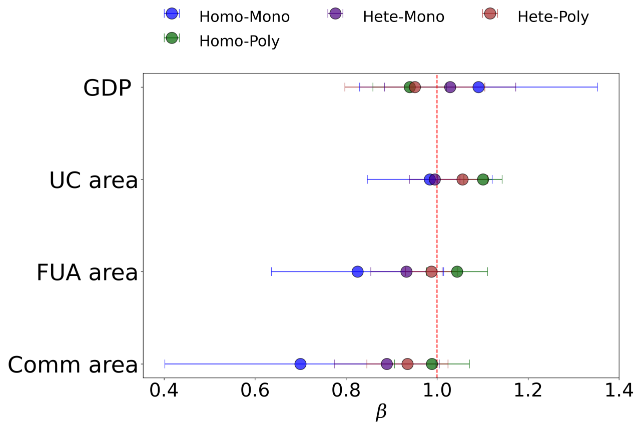

FIG. S3. **Relationship between scaling exponents and spatial organization of the population.** Scaling exponents β of each city class in Fig. S1.

[1] M. Kummu, M. Taka, and J. H. A. Guillaume, Gridded global datasets for gross domestic product and human development index over 1990–2015, Sci. Data **5**, 1 (2018).

[2] S. Freire and M. Pesaresi, GHS population grid, derived from GPW4, multitemporal (1975, 1990, 2000, 2015), European Commission, Joint Research Centre (JRC) (2015).
