# Supplementary material for: Non-trivial relationship between scaling behavior and the spatial organization of GDP in Indonesian cities
Source: PLoS One. 2022 Nov 10;17(11):e0277433. doi: 10.1371/journal.pone.0277433 (PMC9648789; doi:10.1371/journal.pone.0277433)
Supplement: Attachment — Submitted filename: Response to Reviewers.pdf [file pone.0277433.s002.pdf]

Response to the Reviewers

We thank the Reviewers for their efforts to read our manuscript and critical assessment of our work. In the following, we address their concerns point-by-point.

Reviewer #1

We would like to thank Reviewer #1 for the effort to read our manuscript carefully and gave us constructive comments. Please find the following point-to-point responses to Reviewer #1's comments.

Reviewer Comment 1.1 — This paper attempts at establishing relationships between "urban productivity" and the degree of polycentricity in Indonesian urban areas through measuring scaling effects. The text is pleasant to read, the data processing seems to be clean, but I am afraid that its contribution to existing literature remains marginal, not because of the author's work that seems sound enough but because of the uncertainties linked with the data that are used. The text is clearly written and methodology and results are well explained. Illustrations are useful, well designed and clearly identified. Sources are clearly presented but without enough words about their limitations, especially regarding GDP measurement significance and allocation in the spatial grid. Moreover, because of the methodology that was used for building the data base, it is not at all certain that the data that are provided do represent the product of local economic activity.

Reply: We greatly appreciates the time and effort put forth by Reviewer # 1. We acknowledged that in the previous version of the manuscript, our explanation regarding the assumption and the methods of the GDP database that we used is insufficient. The gridded GDP dataset is constructed from the sub-national GDP per capita data and the gridded population density. As a result, the 1 km^2-scale spatial distribution of the GDP is mostly originated from the spatial variation of the population density. However, the spatial variation from the sub-national GDP per-capita is also non-trivial. To clarify this, we repeat our analysis using gridded population density data. Such analysis is written in the supplementary materials in the revised manuscript and is discussed in details in Reviewer Comment 1.3. In addition, we also rewrite our manuscript and emphasize the limitation of our usage of the GDP dataset.

Reviewer Comment 1.2 — To be clear: naming "GDP microstructure" the simple statistical spatial pattern that correspond to the source of data (which is a disaggregation of broad GDP figures according to the population) could be misleading because the term structure may lead to imagine information about the real networks and value chains among the economic stakeholders inside a city.

Reply: We thank Reviewer #1 for pointing this out. We agree with the reviewer. In the revised manuscript, we abandoned the usage of term "*GDP microstructure*" and replaced it with "*spatial organization of GDP*".

Reviewer Comment 1.3 — Second, it is not sure that GDP from the database do measure urban productivity, especially at intra-urban scale, it may represent local income but also reflect the general hypothesis that were used for its estimation.

Reply: We thank Reviewer # 1 for pointing out this critical comment. We acknowledged that this is indeed the limitation of our analysis. As stated earlier, the gridded GDP dataset is constructed from the sub-national GDP per capita data and the gridded population density. To clarify the differences between the GDP data and the population density data, we then repeat our analysis using the gridded population density data in the supplementary materials of the revised manuscript and found differences for Hete-Poly class. Nevertheless, the general trend is similar thus confirming that 1 km^2-scale spatial distribution of the GDP is mostly originated from the spatial variation of the population density. In the revised manuscript, we have written about this limitation in the first paragraph of the discussion.

Reviewer Comment 1.4 — On the theoretical level, I find in the paper a few remarks reminding of the former attempts made decades ago to adapt central place theory that was conceived to describe interurban patterns, to the internal structure of cities. Intra-urban space has a different structure because the socio-spatial processes that differentiate the parts of a city (i.e. within a daily urban system) are not the same as those shaping the functional and spatial differences between more distant cities. Scaling laws were theoretically built for representing interurban relationships, their validity is less well established inside urban areas.

Reply: We thank Reviewer # 1 for this interesting comment. However, the discussion about such intra-urban and inter-urban connections are beyond the scope of this paper. Instead, we are interested in the relationship between intra-urban spatial pattern and scaling behavior.

Reviewer Comment 1.5 — Some conclusions are probably going beyond what the data authorize according to the many uncertainties and weaknesses from the source that do not always represent accurately what is expected. For instance, a sentence like "Regardless of their heterogeneity, hotspots in monocentric cities in Indonesia experience the physical expansions of hotspots that are not followed by the economic growth" should be highly hypothetic.

Reply: We thank Reviewer # 1 for this comment. In the revised manuscript, we acknowledge the limitations and the uncertainty of our analysis. Especially, given the comments 1.1 and 1.3, in discussion part, we added a new paragraph describing the limitation of our data source and a need for further investigation with different data source. In addition, we removed the sentence "Regardless of their heterogeneity, hotspots in monocentric cities in Indonesia experience the physical expansions of hotspots that are not followed by the economic growth" in the revised manuscript.

Reviewer Comment 1.6 — As well the first paragraph of the discussion raises questions without taking care of the real significance of the data.

Reply: We thank Reviewer # 1 for this comment. In the revised manuscript, we discuss the limitations of our analysis first before raising any questions regarding the obtained results.

Reviewer Comment 1.7 — Line 14 The expression "human needs" is not accurate for social scientists. Needs are so highly depending on the wealth and culture of societies, they have so much evolved over time that they have to be considered no longer as human but always socially defined, varying and evolving. According to regional inequalities in Indonesia, it may be that the computed scaling relationship between population and wealth reflect quite heterogeneous local economic situations.

Reply: We thank Reviewer # 1 for this comment. We agree with Reviewer # 1 and replace the term "human needs" with "urban features that tends to be distributed evenly per-capita such as housing and water. We also agree with Reviewer #1 for his/her comments regarding regional inequalities in Indonesia. Such discussion is elaborated in line 86-105 in the revised manuscript, where we observed that the deviation from the fitted power-law has a geographical pattern that represents the Java-centric economy.

Reviewer Comment 1.8 — lines 27-30 "spatial microstructures of cities "should be defined here. Does it have to do with the physical and socio-economic morphology of urban areas? What is the difference with the polycentric structures? Why using a different vocabulary ?

Reply: We thank Reviewer # 1 for this comment. As we discussed earlier in Reviewer Comment 1.2, the term "spatial microstructure" has been replaced with "spatial organization" in the revised manuscript. However, our measure of spatial organization goes beyond the measure of polycentricity or spatial dispersion. In addition the the spatial dispersion, we also take into account the heterogeneity and it is represented by the Gini index. Such explanation is added to line 152-158

Reviewer Comment 1.9 — lines 99-101 very unclear sentence: "This implies that the suppressed fluctuation in higher population regime (Fig. 1) is coupled with the geographical constraint". What do you mean?

Reply: We thank Reviewer # 1 for pointing out this unclear sentence. In the revised manuscript, we have removed such sentence.

Reviewer Comment 1.10 — lines 106-116 take for granted that the sublinear behavior of urban surface with population guaranties "efficiency of growth". This is a biased interpretation of urban spatial structure because it do not take into account the centre-periphery process.

Reply: We thank Reviewer # 1 for pointing this issue out. We agree with the reviewer and removed such statements. Nevertheless, scaling analysis of the city area in this paper are further divided into two kinds of the urban area, namely urban centers (centre) and commuting zone(periphery).
